# A case report of prostate cancer with leptomeningeal metastasis

**DOI:** 10.1002/cnr2.1463

**Published:** 2021-06-05

**Authors:** Mansoureh Dehghani, Babak PeyroShabany, Ramin Shahraini, Danial Fazilat‐Panah, Fateme Hashemi, James S. Welsh, Seyed Alireza Javadinia

**Affiliations:** ^1^ Cancer Research Centre Mashhad University of Medical Sciences Mashhad Iran; ^2^ Department of Internal Medicine, School of Medicine Sabzevar University of Medical Sciences Sabzevar Iran; ^3^ Department of Radiology, School of Medicine Sabzevar University of Medical Sciences Sabzevar Iran; ^4^ Cancer Research Center Babol University of Medical Sciences Babol Iran; ^5^ Department of Internal Medicine Mashhad University of Medical Sciences Mashhad Iran; ^6^ Edward Hines Jr VA Hospital and Loyola University Chicago Stritch School of Medicine Chicago Illinois USA; ^7^ Cellular and Molecular Research Center Sabzevar University of Medical Sciences Sabzevar Iran

**Keywords:** leptomeningeal carcinomatosis, prostate cancer

## Abstract

**Background:**

Prostate cancer is the most prevalent cancer in men. However, leptomeningeal involvement by prostate carcinoma is a rare event.

**Case:**

Here, we report a 69‐year‐old patient with castration‐resistant metastatic prostate cancer who presented with headache and ataxia. Brain MRI revealed a huge invasive interaxial mass at right occipital lobe with diffuse thickening and enhancement of meninges, the arachnoid, and the pia mater, and he was diagnosed with leptomeningeal carcinomatosis. The patient received whole brain radiotherapy.

**Conclusion:**

Despite the fact that brain and leptomeningeal metastases are not very common in patients with prostate cancer, signs and symptoms of nervous system disorders should be assessed carefully, and consideration of such unusual metastases must be considered.

## 1 INTRODUCTION

Leptomeningeal metastases from solid tumors are relatively uncommon events with an extremely poor prognosis. They are more common in breast cancer, lung cancers, and melanoma but can occasionally occur in other cancers. In rare occasions, leptomeningeal carcinomatosis (LC) can occur in patients with prostate cancer.^1^, ^2^ Common extranodal metastatic sites of prostate cancer are bones, liver, and lung, and metastases to the central nervous system (CNS) and leptomeninges are extremely rare.^3^ But as more effective treatments emerge and patients' life expectancies increase, leptomeningeal metastases seem to be increasing and underdiagnosed, and autopsy studies suggest that it has always been more frequent than what is stated.^4^ Treatment of prostate cancer LC is not standardized, and various approaches have been reported, mostly as case studies.^5^ Here, we report a 69‐year‐old patient with metastatic prostate cancer who presented with headache and ataxia and was diagnosed with LC.

## 2 CASE PRESENTATION

A now 69‐year‐old male was diagnosed in 2016 with cT3N10M0 Gleason 5 + 4 = 9 prostate adenocarcinoma. The patient received 6 months of neoadjuvant androgen deprivation therapy (ADT) followed by definitive radiotherapy (70 Gy/35 fractions) along with concurrent ADT. ADT then continued for a total duration of 18 months. Seven months after completion of the ADT, the serum prostate‐specific antigen (PSA) raised; however, there was no evidence of distant metastasis on Tc‐99m bone and whole body computed tomography (CT) scans. Therefore, hormone manipulation by initiating ADT (goserelin acetate implant, 10.8 mg, every 12 weeks) plus an antiandrogen (tablet bicalutamide, 50 mg, daily) were administrated. Despite this treatment, the PSA continued to rise. Consequently, six cycles of docetaxel were administrated [docetaxel (75 mg/m^2^) D1 + prednisolone 5 mg twice daily D1‐21, every 3 weeks]. Due to the lack of response and rising level of PSA, docetaxel was discontinued, and abiraterone acetate (AA) was administrated [AA (1000 mg) D1‐28 + prednisolone 5 mg twice daily D1‐21, every 4 weeks]. Meanwhile, the patient complained of progressive neck pain, and the physical exam demonstrated spinal tenderness. Therefore, a 99mTc‐MDP bone scan and a whole‐body CT scan were ordered and showed widespread bone metastases with no evidence of visceral metastases. Along with AA, zoledronic acid [4 mg, every 28 days] and palliative radiotherapy of the cervical spine were initiated (30 Gy/10 fractions). Three months after the start of AA, the assessment of PSA showed no considerable response, leading to a change in chemotherapy regimen to mitoxantrone. However, there was no clinical or biochemical response to three cycles of the new treatment. Due to lack of access to cabazitaxel and enzalutamide, all treatments were discontinued, and only zoledronic acid was prescribed.

**FIGURE 1:**
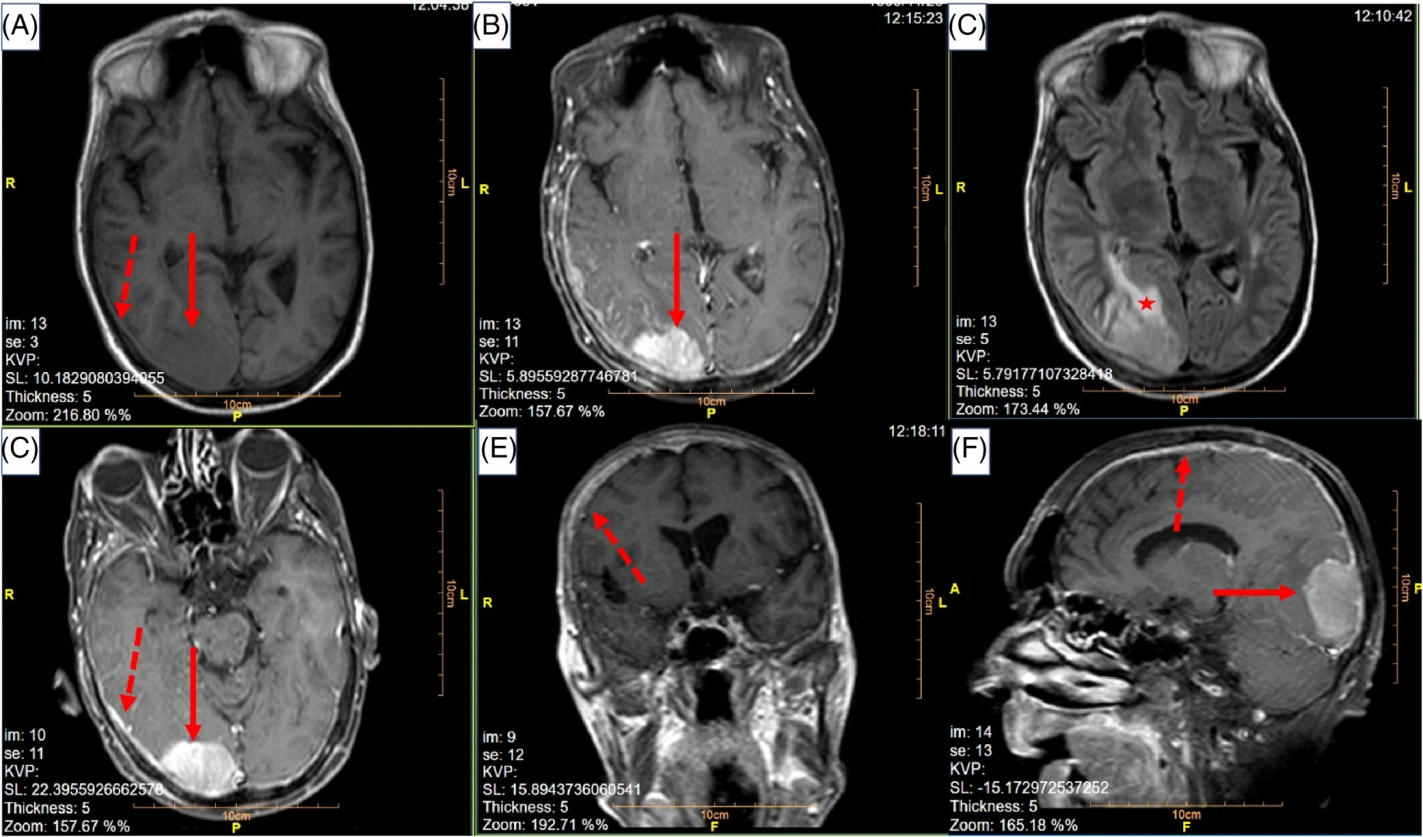
MRI brain obtained from (A) axial plane, T1 without gadolinium contrast, (B) axial plane, T1 with contrast, (C) axial plane, T2, (D) axial plane, T1 with contrast, (E) coronal plane, T1 with contrast, and (F) sagittal plane, T1 with contrast. Solid lines show the 50 × 40 mm interaxial mass at right occipital lobe which was hypo/isosignal at T1‐weighted images and hypersignal at T2‐weighted images, with enhancement of the lesion at contrast‐enhanced T1‐weighted images. Dashed lines show diffuse leptomeningeal enhancement. The star shows the perilesional vasogenic edema

**FIGURE 2:**
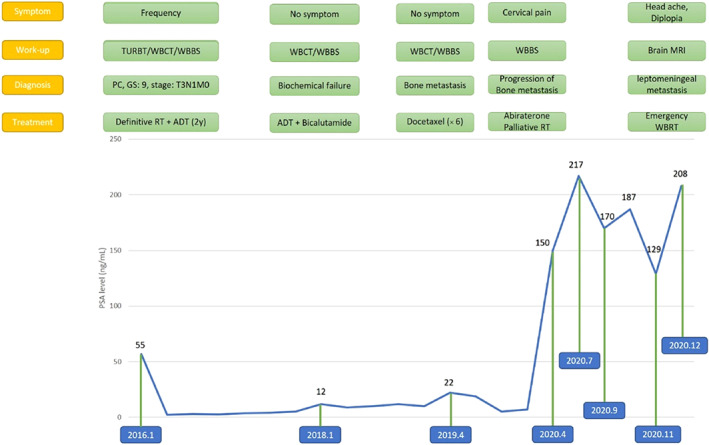
Clinical, biochemical, and treatment course of disease

After 3 months, the patient presented to the clinic complaining about recent headache, true vertigo, impaired vision in the right eye, diplopia, and balance disturbance. These complaints had evolved over the prior 7 days. His Eastern Cooperative Oncology Group (ECOG) performance status was recoded as the grade I. Physical examination showed gait disturbance (wide‐based gait), papilledema, and palsy of nerve III at the right side. At presentation, serum PSA level was 300 ng/mL. Oral dexamethasone 4 mg every 6 h was administered, and in order to evaluate the patient, a brain MRI was ordered. During the next 7 days, the patients' lower extremities became increasingly weak, and eventually, he was unable to walk independently. In addition, overall functional status deteriorated, and ECOG performance status reduced to the grade III. The brain MRI revealed meningeal diffuse enhancement (which was highly suggestive of LC) with a bulky focus of metastatic tumor attached to the dura (Figure 1). The patient was clinically diagnosed with LC originating from prostate cancer based on the history of the aggressive disease, physical examination, and clinical presentation, high level of PSA, and highly suggestive MRI findings. The results, other diagnostic options including cerebrospinal fluid (CSF) studies, and the poor prognosis were discussed with the patient and his family, and then the patient was treated with palliative whole‐brain radiation (30 Gy/10 fractions). Figure 2 shows the courses of disease during 2016–2020. One month after the completion of whole‐brain irradiation, the neurological symptoms progressed leading to the patient's death.

## 3 DISCUSSION

Leptomeningeal carcinomatosis is defined as metastasis of malignant neoplastic disease to the leptomeninges. This event is a rather rare complication of cancer. Leptomeningeal spread of cancerous cells occurs more commonly with hematologic tumors (10%–15%) and less often with solid tumors (1%–5%). Spread of hematologic malignancies to the leptomeninges is often referred to as lymphomatous meningitis, but spread of solid tumors to leptomeninges is referred to as LC, which is relatively more frequent in breast and lung cancer, and malignant melanoma.^1^, ^2^ Common symptoms include headache, seizures, sensory deficits, gait abnormalities, and memory problems. Other possible symptoms are nausea and vomiting, fatigue, pain, incontinence, and confusion. The most commonly affected cranial nerves are III, V, VI, VII, and VIII. Communicating hydrocephalus can be caused by decreased reabsorption of CSF. Clinical examination usually suggests involvement of more than one area of the CNS.^3^

Metastases to the CNS, especially LC, are relatively rare complications of prostate cancer.^5^ A large retrospective study from the MD Anderson Cancer Center (1979–2011) revealed that, out of 41 830 patients with prostate cancer, only 7 (0.016%) had leptomeningeal metastases.^6^ Interestingly, older autopsy‐series publications described a somewhat higher prevalence of CNS involvement in prostate cancer. One such study examined autopsies of 126 patients with prostate cancer between 1954 and 1981 and found that 11.1% had metastases to the CNS, with 9.5% having some involvement of the dura.^7^ Additionally, some researchers have suggested that the incidence of CNS involvement may be rising in prostate cancer patients due to new therapies and prolonged survival.^4^ It has even been suggested that the discrepancy between the systemic effectiveness of docetaxel and its low CNS penetration may also contribute to the potential rise in prostate cancer LC.^8^

The advances in MRI techniques have greatly aided the diagnosis of LC, and specific MRI findings are considered highly suggestive. However, a definitive pathologic confirmation requires CSF cytology (sometimes more than once) or meningeal biopsy, which is not usually performed premortem. The false‐negative rate for CSF cytology for all tumor types is 50% with a single lumbar tap, 20% with two, and 10% with three.^9^ Because of these statistics and due to the invasive procedures required for these confirmatory tests, it is important to decide whether such procedures are absolutely necessary when diagnostic imaging and clinical findings are consistent with LC diagnosis.

The diagnostic method of LC in prostate cancer has evolved over the years.^5^ Currently, MRI with contrast is considered the most sensitive imaging modality, with almost 100% sensitivity. CT scans have a significantly reduced sensitivity of only 26%–56%. Gadolinium‐enhanced T1‐weighted MRI sequences are considered the best noninvasive means of detecting LC. Diagnostic findings include leptomeningeal enhancement of the brain, spinal cord, cauda equina, or sub‐ependymal areas. The enhancement may extend into the sulci of the cerebrum or folia of the cerebellum. Other suggestive findings are minimal enhancement of the aforementioned areas, cranial nerve enhancement, superficial cerebral lesions, and communicating hydrocephalus.^10^ Experts suggest that when there is strong evidence of LC on MRI, cytological confirmation is not necessary, and physicians can proceed with the treatment.^5^, ^11^

There is currently no standard treatment for prostate cancer LC, and treatment options are largely based on expert opinion and local practices, given the fact that after discussing the prognosis and uncertain options, many patients and families collectively elect to withdraw care, as it is reported in previous case reports.^12^

One retrospective study of prostate and other genitourinary cancer patients with LC from the MD Anderson Cancer Center identified 31 patients with this condition. Out of 31 patients, 11 received both intrathecal chemotherapy (methotrexate, cytarabine, or topotecan) and radiation therapy, five patients received only intrathecal chemotherapy, seven patients received only radiation therapy, and eight patients did not receive either. No significant difference in survival was observed between the groups.^6^ Other treatment options suggested by the literature include hormonal treatment (in castration‐sensitive prostate cancer), corticosteroids, and debulking surgery. However, all of these treatments have been associated with poor outcomes.^3^, ^13^ In a review in 2017, the authors documented the different attempted treatments for leptomengeal metastases of different origins. All the reported trials provided palliation at best; no curative options have emerged. The authors concluded that a variety of treatment modalities, such as intrathecal chemotherapy and radiation therapy, may improve median survival from 4–6 weeks to 3–6 months.^14^ At this time, one main goal of the treatment strategies is to provide tumor‐specific intrathecal therapies. However, due to toxicities and the aggressive biology of tumors that invade the meninges and epidural space, more studies are required to select the treatment of choice for these events. Also because of poor prognosis and mostly poor performance of prostate cancer patients suffering from LC, some researchers have suggested that the treatment approach should include a palliative care referral as soon as the diagnosis is made.^6^

The prognosis is generally poor after the diagnosis of LC. A literature review of 14 case studies noted that 12 of these patients survived 1 month or less following the diagnosis, one patient survived 5 months, and one patient survived more than 16 months.^13^ Factors such as preserved cognition, controlled systemic disease, normal CSF glucose, and low CSF protein are found to be associated with slightly longer survival^6^; however, the main points that oncologists need to take into consideration are that as more effective treatments emerge and increased life expectancy is achieved, more and more patients are anticipated to be diagnosed with leptomeningeal disease, early diagnosis is important in order to administer the appropriate treatment and avoid permanent neurological deficits, and the treatment should be individualized, depending on each patients' presentation and performance.^3^

## CONFLICT OF INTEREST

There was no conflict of interest to be reported.

## AUTHOR CONTRIBUTIONS

All authors had full access to the data in the study and take responsibility for the integrity of the data and the accuracy of the data analysis. *Conceptualization*, M.D., B.P., F.H.; *Methodology*, B.P., R.S., F.H.; *Investigation*, M.D., B.P., F.H.; *Formal Analysis*, M.D.; Writing—Original Draft, R.S., D.F.‐P., J.S.W.; Writing—Review & Editing, R.S., D.F.‐P., J.S.W.; *Supervision*, D.F.‐P.; *Data Curation*, M.D., B.P., F.H.; *Project Administration*, R.S.; *Validation*, D.F.‐P., J.S.W.

## ETHICAL STATEMENT

Written informed consent was obtained from the participant and approvals from concerned review boards/committees (human) are documented.

## Data Availability

The data that support the findings of this study are available on request from the corresponding author. The data are not publicly available due to privacy or ethical restrictions.

## References

[1] Wang N , Bertalan MS , Brastianos PK . Leptomeningeal metastasis from systemic cancer: review and update on management. Cancer. 2018;124(1):21‐35. 10.1002/cncr.30911. 29165794 PMC7418844

[2] Grossman SA , Krabak MJ . Leptomeningeal carcinomatosis. Cancer Treat Rev. 1999;25(2):103‐119. 10.1053/ctrv.1999.0119. 10395835

[3] Orphanos G , Ardavanis A . Leptomeningeal metastases from prostate cancer: an emerging clinical conundrum. Clin Exp Metastasis. 2010;27(1):19‐23. 10.1007/s10585-009-9298-z. 19904616

[4] Lin C , Turner S , Gurney H , et al. Increased detections of leptomeningeal presentations in men with hormone refractory prostate cancer: An effect of improved systemic therapy?. J Med Imaging Radiat Oncol. 2008;52(4):376‐381. 10.1111/j.1440-1673.2008.01973.x. 18811763

[5] Neeman E , Salamon N , Rettig M . Leptomeningeal carcinomatosis of prostate cancer: a case report and review of the literature. Rev Urol. 2020;22(2):80‐84. 32760233 PMC7393686

[6] Yust‐Katz S , Mathis S , Groves MD . Leptomeningeal metastases from genitourinary cancer: the University of Texas MD Anderson Cancer Center experience. Med Oncol. 2013;30(1):429. 23292836 10.1007/s12032-012-0429-z

[7] Lefkowitz M , Coggin JT , Skoog SJ , et al. Intracranial metastases in prostate cancer. Cancer. 1984;53(12):2728‐2730. 10.1002/1097-0142(19840615)53:12<2728::aid-cncr2820531231>3.0.co;2-x. 6722732

[8] Caffo O , Gernone A , Ortega C , et al. Central nervous system metastases from castration‐resistant prostate cancer in the docetaxel era. J Neurooncol. 2012;107(1):191‐196. 21989810 10.1007/s11060-011-0734-y

[9] Glantz MJ , Cole BF , Glantz LK , et al. Cerebrospinal fluid cytology in patients with cancer. Cancer. 1998;82(4):733‐739. 10.1002/(sici)1097-0142(19980215)82:4<733::aid-cncr17>3.0.co;2-z. 9477107

[10] DeAngelis LM , Boutros D . Leptomeningeal metastasis. Cancer Invest. 2005;23(2):145‐154. https://pubmed.ncbi.nlm.nih.gov/15813508/. 15813508

[11] Bernstein WB , Kemp JD , Kim GS , et al. Diagnosing leptomeningeal carcinomatosis with negative CSF cytology in advanced prostate cancer. J Clin Oncol. 2008;26(19):3281‐3284. 18591564 10.1200/JCO.2008.16.4533

[12] Carroll RD , Leigh EC , Curtis Z , et al. A case of leptomeningeal carcinomatosis from aggressive metastatic prostate cancer. Case Reports in Oncology. 2019;12(1):311‐316. 10.1159/000499761. 31123457 PMC6514516

[13] Cone LA , Koochek K , Henager HA , et al. Leptomeningeal carcinomatosis in a patient with metastatic prostate cancer: case report and literature review. Surg Neurol. 2006;65(4):372‐375. 16531199 10.1016/j.surneu.2005.08.026

[14] Nayar G , Ejikeme T , Chongsathidkiet P , et al. Leptomeningeal disease: current diagnostic and therapeutic strategies. Oncotarget. 2017;8(42):73312‐73328. 29069871 10.18632/oncotarget.20272 PMC5641214

